# Multiferroic coreshell magnetoelectric nanoparticles as NMR sensitive nanoprobes for cancer cell detection

**DOI:** 10.1038/s41598-017-01647-x

**Published:** 2017-05-09

**Authors:** Abhignyan Nagesetti, Alexandra Rodzinski, Emmanuel Stimphil, Tiffanie Stewart, Chooda Khanal, Ping Wang, Rakesh Guduru, Ping Liang, Irina Agoulnik, Jeffrey Horstmyer, Sakhrat Khizroev

**Affiliations:** 1 Department of Electrical Engineering Florida International University, Miami, Florida 33174 USA; 2 Herbert Wertheim College of Medicine, Florida International University, Miami, Florida 33199 USA; 3 Electrical and Computer Engineering, University of California, Riverside, CA 92506 USA; 4 Neuroscience Centers of Florida Foundation, Miami, FL 33133 USA

## Abstract

Magnetoelectric (ME) nanoparticles (MENs) intrinsically couple magnetic and electric fields. Using them as nuclear magnetic resonance (NMR) sensitive nanoprobes adds another dimension for NMR detection of biological cells based on the cell type and corresponding particle association with the cell. Based on ME property, for the first time we show that MENs can distinguish different cancer cells among themselves as well as from their normal counterparts. The core-shell nanoparticles are 30 nm in size and were not superparamagnetic. Due to presence of the ME effect, these nanoparticles can significantly enhance the electric field configuration on the cell membrane which serves as a signature characteristic depending on the cancer cell type and progression stage. This was clearly observed by a significant change in the NMR absorption spectra of cells incubated with MENs. In contrast, conventional cobalt ferrite magnetic nanoparticles (MNPs) did not show any change in the NMR absorption spectra. We conclude that different membrane properties of cells which result in distinct MEN organization and the minimization of electrical energy due to particle binding to the cells contribute to the NMR signal. The nanoprobe based NMR spectroscopy has the potential to enable rapid screening of cancers and impact next-generation cancer diagnostic exams.

## Introduction

Rapid identification of cancer cells is vital for cancer prevention and treatment. Traditional techniques which rely on biochemical staining require a tedious sample preparation and are limited to a few biomarkers. A more advanced approach based on polymerase chain reaction (PCR) remains cost-ineffective in a small-clinic environment. Therefore, recently there has been increased interest in magnetic nanoparticle bio sensing (MNB). Due to a new dimensionality provided by the presence of externally-controlled magnetic moments, MNB promises to enable high-specificity screening and fast diagnostic of pathogens^1^. Indeed, one could envision an apparatus in which magnetic nanoparticles are used to couple intrinsic information related to single cells, (e.g. the electric charge profile on the surface of the cell membrane in a specific biological microenvironment) to an external magnetic device such as a nuclear magnetic resonance (NMR) system. However, the current progress in this area still remains relatively slow. The main challenge is to couple magnetic nanoparticles to intrinsic information at the cellular or intra-cellular level with sufficiently high efficacy to be able to process the information with a magnetic detection system. While the system measures magnetic fields, the intrinsic cellular information is reflected in electric fields^2^. It can be noted that in the cellular microenvironment, each cell structure, corresponding to a specific cancer type and cancer progression stage, is characterized by a certain membrane surface morphology which in turn results in a signature electric-field configuration^3–5^. However, traditional conventional magnetic nanoparticles would not be able to detect this complex electric-field configuration unless they have intrinsically connected electric charges. To address this problem, in lieu of the traditional magnetic nanoparticles, we have used a new type of multiferroic nanostructures known as magnetoelectric nanoparticles (MENs)^6–9^. Unlike the traditional magnetic nanoparticles, MENs have both magnetic and electric dipole moments; additionally, these two different moments are correlated through the magnetoelectric (ME) effect^10–12^. Due to the presence of an electric charge, MENs preferentially attach to cell-specific sites and thus provide access to intrinsic information at the subcellular level. Simultaneously, due to the presence of the ME effect, MENs allow the conversion of this intrinsic electric field information into a specific magnetic field pattern which in turn could be measured through a magnetic measurement setup such as a NMR system. Because each cell type has its own signature electric field distribution either at the membrane or at the intracellular level, such NMR measurements could be used to distinguish different cell types from each other at the subcellular level.

## Results

In this study, for comparison, MENs with a relatively strong ME effect and traditional magnetic nanoparticles (MNPs) without any ME effect were integrated into the media with different cancer cell lines and then the media’s NMR spectra were measured under equivalent conditions. Specifically, the mean diameter of coreshell CoFe_2_O_4_@BaTiO_3_ MENs (30-nm) was 30 ± 6 nm and that of the ferrimagnetic CoFe_2_O_4_ spinel core was 15.2 ± 4.0 nm (15-nm). The same 15 nm ferrimagnetic core nanostructures, without the perovskite (BaTiO_3_) shell, were used as MNPs.

**Figure 1:**
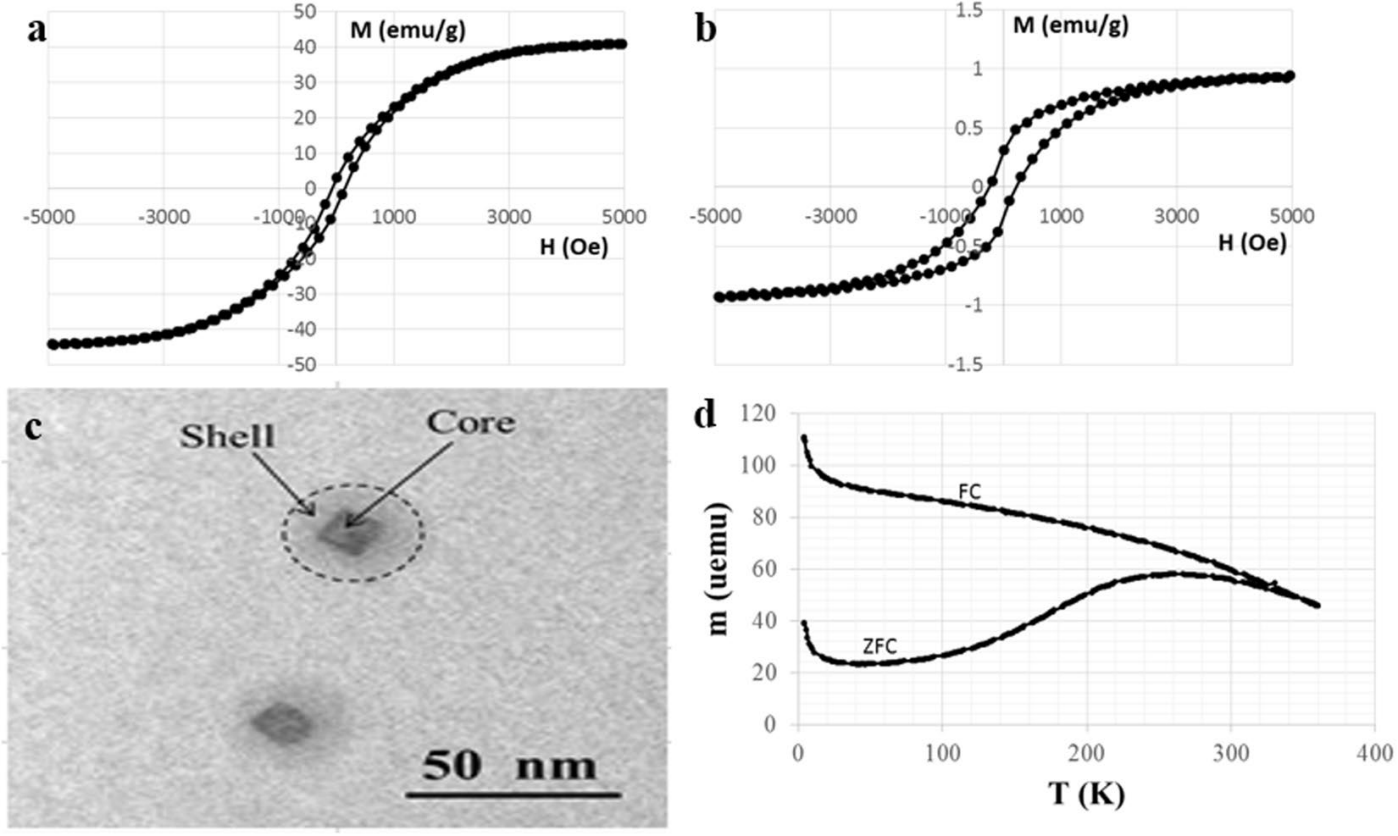
Room-temperature M-H hysteresis loops of (**a**) 30-nm MENs and (**b**) 15-nm MNPs. (**c**) Shows A TEM image showing a coreshell structure of 30-nm MENs. (**d**) Blocking temperature measurement curves including zero-field cooling (ZFC) and field cooling (FC) curves in a field of 100 Oe.

Figure 1a and b show room-temperature M-H hysteresis loops of MENs and MNPs, respectively, measured via a vibrating sample magnetometry (VSM) system Lakeshore 7300. Despite the fact that the magnetic components in the two cases were equivalent, according to the magnetic hysteresis loops, MENs and MNPs had saturation magnetizations of approximately 1 and 40 emu g^−1^, respectively, and coercivity fields of approximately 310 and 90 Oe, respectively. To understand the MENs’ temperature dependence and measure the transition into the superparamagnetic mode, typical M-H hysteresis loops of MENs in a temperature range from 4 to 300 K were obtained via a cryogenic vibrating sample magnetometer Quantum Design PPMS. The standard magnetization versus temperature curves under zero field cooling (ZFC) and non-zero field cooling (FC) conditions that determine the blocking temperature are shown in Fig. 1d. The blocking temperature is the temperature above which the nanoparticles become superparamagnetic. In this case, it is above 300 K, which confirms that the magnetic cores of MENs do not become superparamagnetic at room temperature despite their small size. The ME coefficient, α, for these nanostructures has been previously measured to be in the range from 10 to over 100 mV cm^−1^ Oe^−1^ ^13^_._

**Figure 2:**
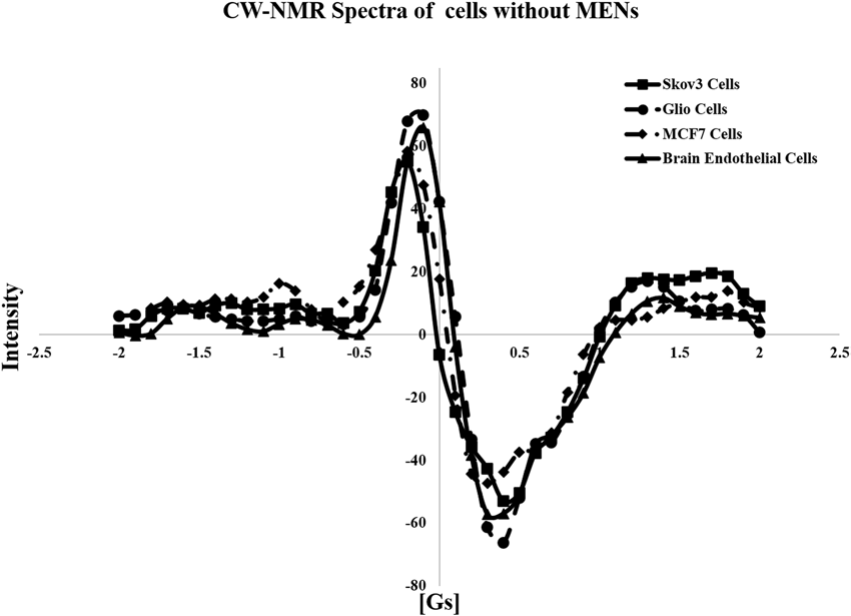
CW-NMR spectra of cell media without MENs for four types of cells: (i) ovarian carcinoma cells Skov3, (ii) glioblastoma cells U87-MG, and (iii) breast adenocarcinoma cells MCF-7, respectively, and (iv) normal brain endothelial cells.

Figure 2 shows continuous wave NMR (CW-NMR) spectra for three cancer cell lines under study, including ovarian carcinoma cells Skov3, glioblastoma cells U87-MG, and breast adenocarcinoma cells MCF-7, respectively, and normal brain endothelial cells without any nanoparticles being present in either one of the media. The NMR spectrum, representing a chemical shift due to intrinsic molecular interactions, is measured as an absorption energy in the field sweep range from −5 to +5G and at a frequency of 14,000 KHz. It can be noted that the four cell lines do not significantly differ from each other.

**Figure 3:**
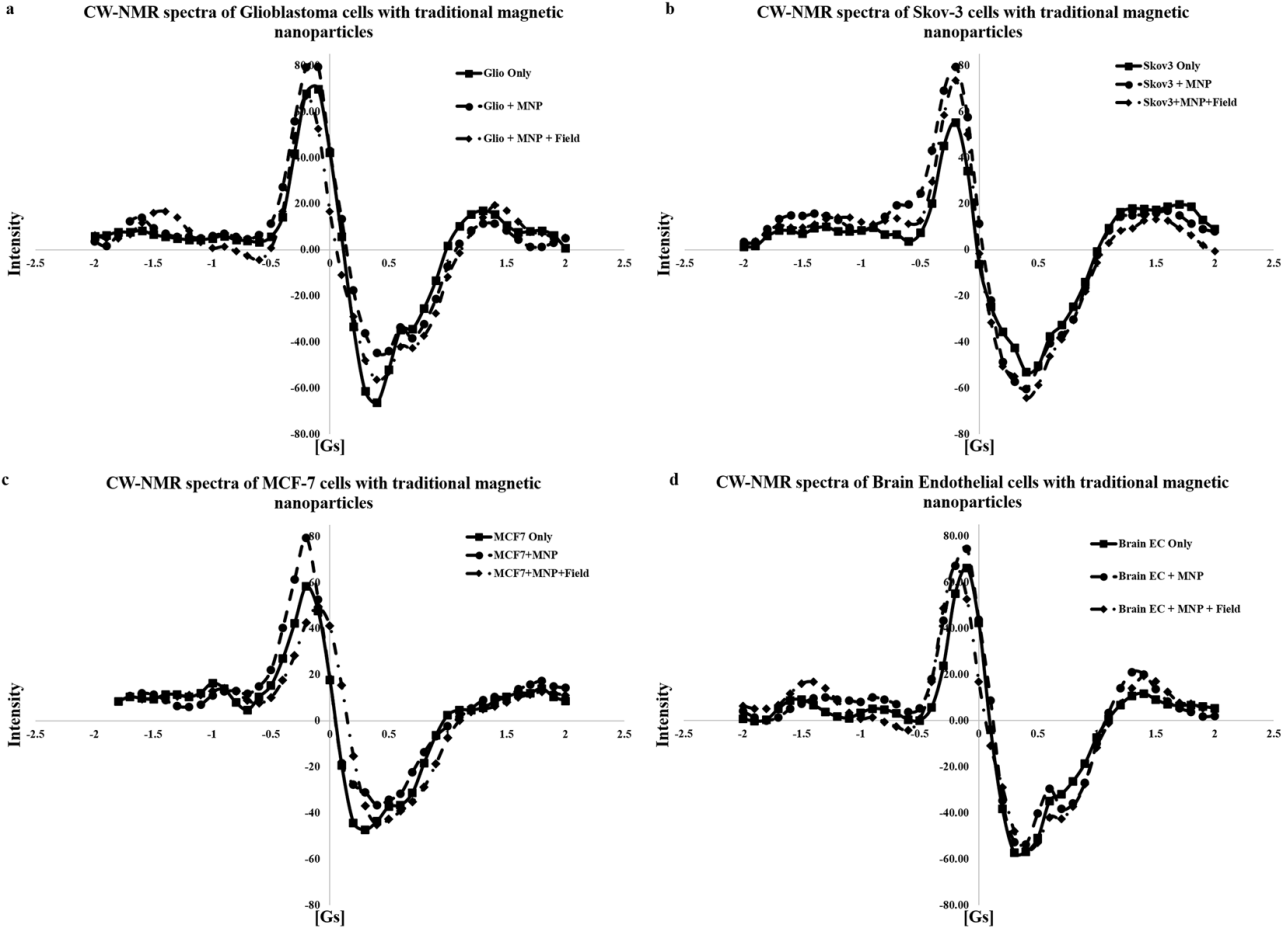
CW-NMR spectra including cells only, cells incubated with MNPs without and with application of a 100-Oe d.c. field for (**a**) glioblastoma cells U87-MG, (**b**) ovarian carcinoma cells Skov3, (**c**) breast adenocarcinoma cells MCF-7, respectively, and (**d**) normal brain endothelial cells.

Figure 3a–d show four sets of CW-NMR spectra of media obtained by incubating the above three cancer cell lines and one non-cancerous normal cell line, respectively, for 15 hours with traditional MNPs. Each set consists of three curves including spectra for cells only, cells incubated with MNPs without the application of an external field, and cells incubated with MNPs under application of a 100 Oe d.c field. The concentration of the nanoparticles in each media was approximately 150 μg ml^−1^. Similar to the case without any nanoparticles, neither of the spectra (i.e. cells + MNPs and cells + MNPs + Field) for the four cell lines significantly differs from the corresponding cell line without MNPs incubation as well as between each other.

**Figure 4:**
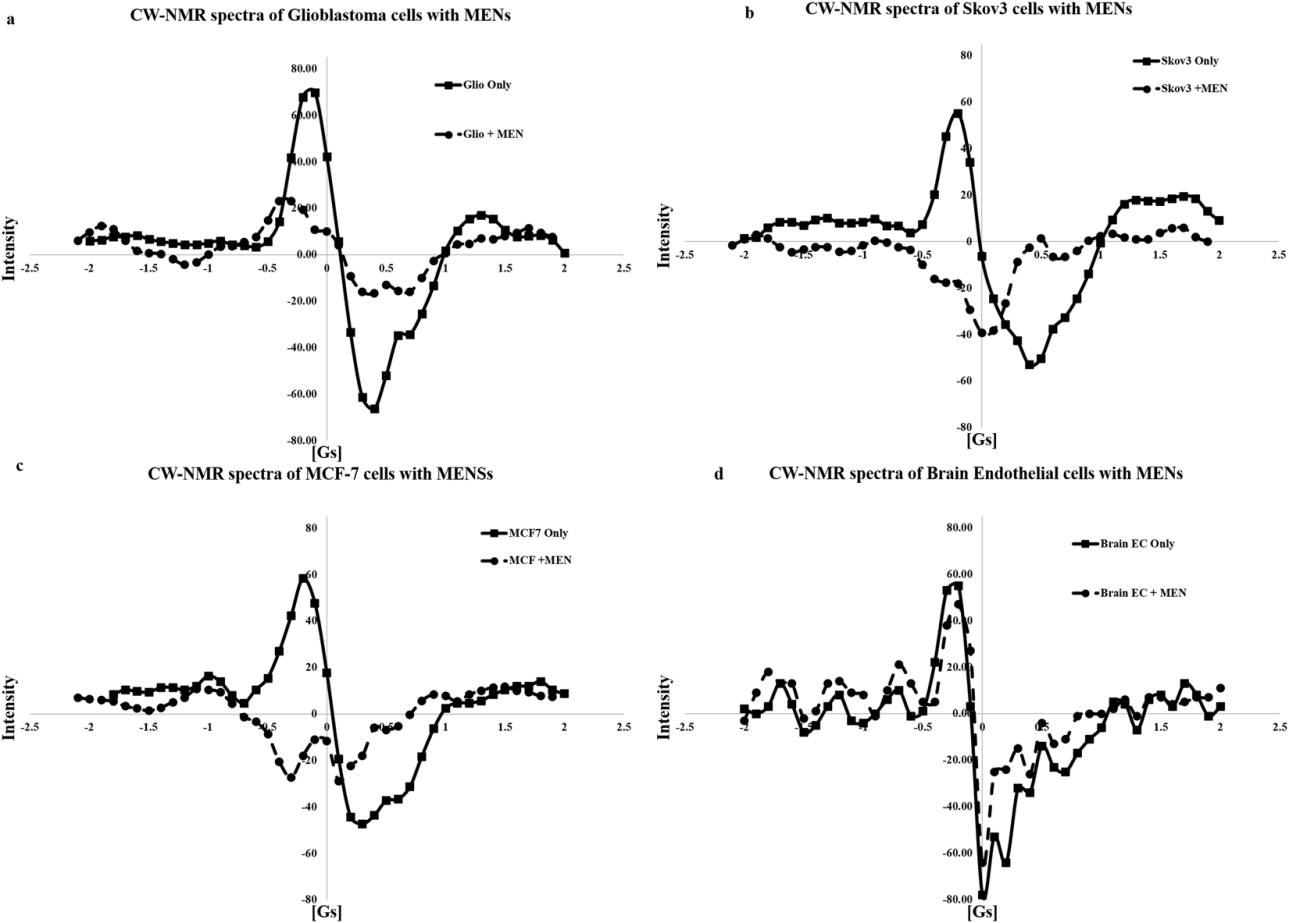
CW-NMR spectra including cells only, cells incubated with MENs for (**a**) glioblastoma cells U87-MG, (**b**) ovarian carcinoma cells Skov3, (**c**) breast adenocarcinoma cells MCF-7, respectively, and (**d**) normal brain endothelial cells.

**Figure 5:**
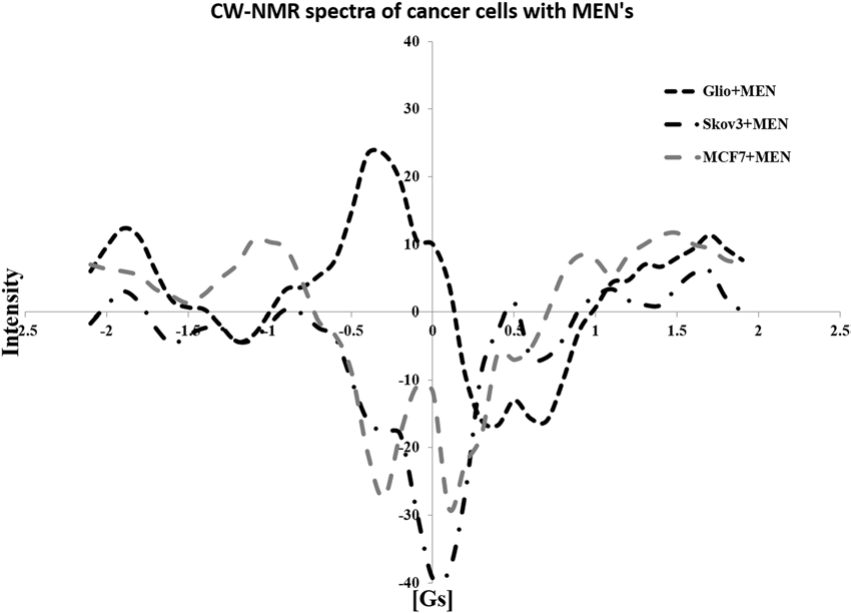
CW-NMR spectra of cell media with MENs for cancer cells.

However, this trend drastically changes if MENs are used instead of MNPs. Figure 4 shows CW-NMR spectra obtained by incubating the same three cancer cell lines and non-cancerous cell line for the same amount of time of 15 hours, with the only exception of having MENs instead of MNPs at the same concentration of approximately 150 μg ml^−1^. According to these spectra, in great contrast to the traditional MNPs, MENs significantly affect the NMR spectrum for each cancer cell type. The only exception is the non-cancerous endothelial cell line; as MNPs, MENs barely affected the spectrum. For comparison, Fig. 5 shows NMR spectra for the same three cancer cell lines incubated with MENs without field application under equivalent conditions with the nanoparticle concentration of approximately 150 μg ml^−1^. Again, unlike the previous case with the traditional MNPs, the NMR spectra for the three cancer cell lines are very different from each other as much as they are different from their normal counterparts. It can be noted that the difference between the spectra is not just quantitative but rather qualitative. Each cell type displays a distinguished set of peaks in its spectrum, thus indicating an intrinsic interaction between MENs and cells.

**Figure 6:**
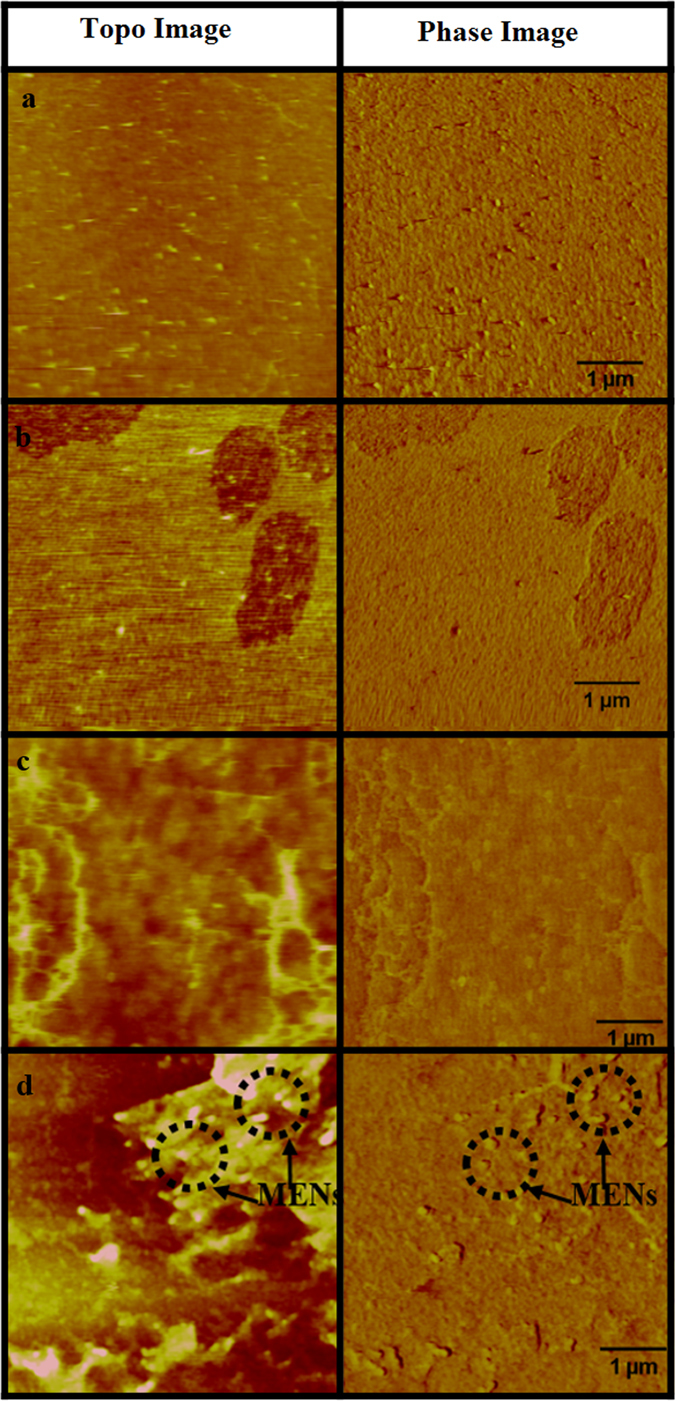
AFM images of the membrane surfaces of (**a**) normal brain endothelial cells, (**b**) normal brain endothelial cells with MENs at a saturated concentration (**c**) glioblastoma cells and (**d**) glioblastoma cells with MENs at a saturated concentration level. Each pair of AFM scans represent z-height and phase images, respectively. The nanoparticles are circled in dotted lines.

Figure 6a–d show atomic force microscopy (AFM) images of the membrane surfaces of normal brain endothelial cells with nanoparticles (a,b), brain endothelial cells with/without nanoparticles, and glioblastoma cells in the presence of MENs (c,d), respectively. Each pair of scans represent z-height and phase images, respectively. The nanoparticles, represented by circled dotted lines, can be seen only in the glioblastoma images. It can be noted that the membranes of normal endothelial cells have a more continuous surface, unlike the membranes of glioblastoma cells which have clearly visible membrane striations with a characteristic size on the order of 100 nm or smaller. MENs seem to be associated with the striations.

## Discussion

Cellular uptake of nanoparticles is a thermodynamic phenomenon that progresses from nanoparticle adsorption on the cell membrane followed by membrane wrapping and invagination in order to minimize the chemical/binding energy^14^. It is important to reiterate that MENs shouldn’t be confused with traditional MNPs. Unlike MNPs, MENs have an electric dipole moment which is proportional to an external magnetic field. As seen from M-H loops, the magnetic moment of MENs is almost two orders of magnitude smaller than that for equivalent MNPs. The fact that despite their significantly smaller magnetic moment, MENs have such a strong effect on the NMR response triggered by an external magnetic field indicate the importance of the magnetic-field-dependent intrinsic electric field around MENs compared to MNPs. When MENs are added into the strongly electrically polarized electric system of the cellular microenvironment, due to the presence of these local electric fields, the energy is further minimized when the MENs bind to cell-specific membrane sites with pronounced electric fields. In case of cancer cells, these sites could be the membrane sites where the local electric fields are enhanced because of edge effects^2,15^. Furthermore, depending on the binding sites or uptake mechanism, the intrinsic electric fields are affected in a specific way due to the electrostatic and chemical bonds at these sites. Again, because, unlike traditional MNPs, MENs have a non-zero electric dipole, electrostatic bonds between MENs and cells can be particularly strong. Here, it is worth mentioning that MENs were shown to acquire a non-zero surface charge due to the double-layer chemistry in the cellular microenvironment^16^. Due to the ME effect, the resulting change in the electric field triggers a change in the magnetic moment of the nanoparticle, which in turn induces a change of the local magnetic field at this particular location. Since the concentration of nanoparticles in the extracellular medium is very high compared to the number of cells, the adsorption of nanoparticles on cell membrane is saturated at long periods of incubation^17,18^. The AFM images above indeed show that MENs are attached to the membrane of glioblastoma cells. The distinct organization of MENs around glioblastoma and brain EC cells was further verified by fluorescence images of these cells incubated with fluorescein isothiocyanate loaded MENs (Supplementary Figure 1S). For nanoparticles with a negative zeta potential, the cellular uptake is strongly dependent on the cell type. The organization of nanoparticles on the membrane surface also depends on the cell type and the mode of uptake (endocytosis, micropinocytosis, etc.)^19^. Therefore, this would change the local net magnetic field in a very specific way depending on all the aforementioned cellular properties. Below, a simple analysis is presented to quantify the resulting change in the local net magnetic field, which in turn is observed as the appearance of new shifts in the NMR spectrum.

Each binding site contributes to the net NMR signal; the contribution, i.e. the electromagnetic energy absorption at this site, is generated when the following resonance condition is satisfied: *hω* = *S*_*n*_ *H*_*net*_, where *h* is the Plank constant, *ω* is the frequency of the electromagnetic wave, *S*_*n*_ is the magnetic moment of the measured nuclear spin (in this study, the proton spin); *H*_*net*_ is the net magnetic field at this location. This local net field is made of the two contributions, (i) the external field generated by the NMR magnets, *H*_*0*_, and (ii) the field due to the ME effect of MENs at the site, *H*_*ME*_, respectively:

$${H}_{net}={H}_{0}+{H}_{ME}.$$

To a zeroth approximation, in the vicinity of a nanoparticle, *H*_*ME*_~Δ*M*_*S*_ = *αE*, where Δ*M*_*S*_ is the nanoparticle’s saturation magnetization change, *α* is the ME coefficient, and *E* is the electric field in the vicinity of the nanoparticle. In turn, this electric field depends on the cell membrane morphology. According to this logic, due to the non-zero ME effect, the measured spectrum shifts on the order of 1 Oe should reflect contributions of MENs from different binding sites (Fig. 3). This is the reason for a significant dependence on the cell type and indeed the resulting changes of spectral shifts are on the order of 1 Oe. It also follows that the traditional purely magnetic nanoparticles, i.e. MNPs, which do not display any ME effect, could not provide this intrinsic contribution specific to each cell type, despite the fact that their saturation magnetization is almost two orders of magnitude higher than that for MENs (Fig. 4). To evaluate the approximate value of the average magnetoelectrically induced electric field, *E*, that holds MENs attached to the cell and thus results in the observed spectrum shift on the order of 1 Oe, we can assume *α*~100 mV Oe^−1^ cm^−1^, Δ*M*_*S*_~1 emu cc^−1^. Then, *E*~10 V cm^−1^. In summary, the above comparison indicates that it is due to the ME effect that the electric properties of cells are crucial in providing the observed signature NMR spectra. The AFM images also confirm this hypothesis. MENs show distinct association with the striations on the glioblastoma cell surface which are on the order of 100 nm (Fig. 6). When the nanoparticle uptake by cells is saturated, approximately 20% of all the nanoparticles are bound to the membrane and thus become visible through AFM imaging^20^. Indeed, brain ECs and glioblastoma cells have very different membrane morphologies resulting in different uptake pathways of nanoparticles. In general, the lipid membrane of cells is comprised of ^1^H-NMR visible fatty acyl chains of triglycerides, free fatty acids and cholesteryl esters^21^. Furthermore, ^1^H-NMR resonant lipid droplets have been found in the cell cytoplasm of brain astrocytomas^22^. Due to the presence of NMR resonant lipid droplets, microdomains have been shown to have a strong correlation with cell signaling, structure^23^ and disease progression^24,25^. It is also worth noting that Hakumaki *et al*. found that healthy brain tissue did not show ^1^H-NMR resonance^21^. According to the current measurements, using MENs significantly enhances the ability of NMR to distinguish between normal and cancer cells as well as between different cancer cells. Indeed, the NMR spectrum of healthy brain endothelial cells was not significantly affected by introducing MENs, compared to the other malignant cell lines. According to the hypothesis, with the introduction of MENs in a saturated state, the relative modification of the averaged NMR energy could be evaluated using a trivial expression; W_MENs_~*αES*_*n*_ *A*, where *A* is a constant between 0 and 1 which represents the relative surface area covered by the striations, which in turn strongly depends on the cancer cell type and the cancer progression stage.

**Figure 7:**
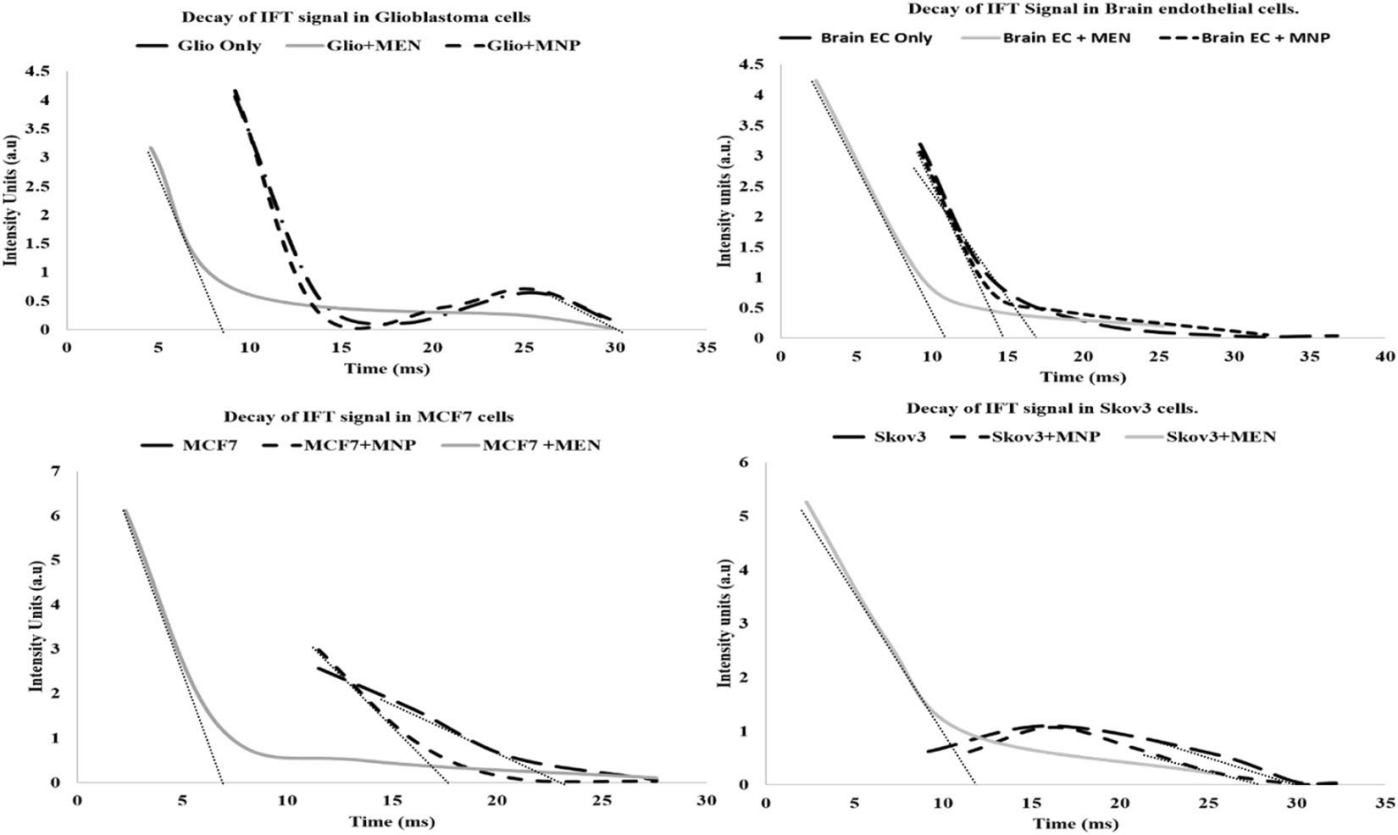
Inverse Fourier Transform (IFT) representation of CW-NMR spectra for Glioblastoma Brain EC, Skov3 and MCF7 with MENs and MNPs. The straight dotted slope lines indicate the approximate equilibration time for each type of measurement.

To more directly represent the observed energy dynamics in the measured NMR spectra, an inverse Fourier Transform (IFT) operation was performed on the spectra. It can be noted that a CW-NMR spectrum represents a signal in the frequency domain while IFT curve represents the same signal in the time domain; in other words, an IFT curve reflects the time dynamics of the NMR-associated energy transfer. The decaying IFT amplitudes for the cell lines under study are shown in Fig. 7. The decay of the IFT curves clearly show how distinguished the spectra for all the cell lines under study are, particularly in the presence of MENs. For comparison, in the presence of MNPs, the IFT curves for the same cells are barely distinguishable from those for the cell lines without nanoparticles. It can be noted that the time dynamic doesn’t change when cells are incubated with MNPs while the NMR-associated energy transfer process for establishing an equilibrium is at least 5 to 20 ms faster when cells are incubated with MENs based on the cell type, as summarized in Table 1.

**Table 1:** Characteristic time constants for different cell-nanoparticle combinations.

	Cells Without Nps	With MNPs	With MENs
Glioblastoma	32 ms	32 b ms	8 ms
Brain endothelial	19 ms	18 ms	11 ms
Breast cancer	24 ms	20 ms	7 ms
Ovarian cancer	32 ms	31 ms	12 ms

## Experimental Section

### Magnetoelectric nanoparticle preparation and characterization

CoFe_2_O_4_-BaTiO_3_ core shell MENs were prepared according to the following procedure. As the first step, the cores of CoFe_2_O_4_ were prepared by the standard hydrothermal method, according to which 0.058 g of cobalt (II) nitrate hexahydrate Co(NO_3_)_2_.6H_2_0 and 0.16 g of Iron (III) nitrate nonahydrate Fe(NO_3_)_3_.9H_2_0 were dissolved in 15 ml of distilled water and 0.2 g of polyvinylpyrrolidone was dissolved in 5 ml of aqueous solution containing 0.9 g of sodium borohydride at 120 °C for 12 hours. For shell growth around the cores, a precursor solution of BaTiO_3_ was prepared by mixing 30 ml of aqueous solution containing 0.029 g of BaCO_3_ and 0.1 g of citric acid with 30 ml of ethanolic solution containing 1 g of citric acid and 0.048 ml of titanium (IV) isopropoxide. Coreshell CoFe_2_O_4_-BaTiO_3_ MENs were prepared by mixing 0.1 g of CoFe_2_O_4_ nanoparticles in the BaTiO_3_ precursor solution and the mixture was sonicated for 2 hrs. Once the CoFe_2_O_4_ nanoparticles were thoroughly dispersed, the mixture was dried on the hot plate at 60 °C overnight while being continuously stirred. The dried powder was heated to 780 °C for 5 hrs. in a furnace (CMF-1100) and cooled at 5 °C per minute to obtain coreshell MENs of ~30 nm diameter. The particles size distribution was measured using dynamic light scattering method (Malvern-Zetasizer) and through transmission electron microscopy.

### Cell culture and sample preparation for CW-NMR

All *in-vitro* cell experiments and biological material handling were approved and performed in accordance within the set guidelines of Florida International University. Three cancerous cell lines including Skov3 (Ovarian adenocarcinoma) (ATCC; Manassas, VA), U87-MG (Glioblastoma) (ATCC), MCF-7A (Breast adenocarcinoma) (ATCC), respectively, and two non-cancerous cell lines including brain endothelial cells (Brain EC, ATCC) and rat smooth muscle endothelial cells (RSMC, ATCC), respectively, were cultured at 37 °C as per manufacturer’s instructions. For nanoparticle studies, cells were detached using 0.25% trypsin solution, plated in 6 well plates and allowed to grow to 80% confluency. MENs were resuspended in cell culture media through sonication and were incubated for 30 minutes. MENs were added to each well at a concentration of 150 μg/ml and the cells were further incubated for 15 hours, to allow attachment of MENs with the cells. Additionally, all the cell lines were incubated with 150 μg/ml traditional MNP (CoFe_2_O_4_) for 15 hours. In order to increase the interaction of MNP’s with cell membrane a d.c magnetic field was also applied. Cells incubated with MNPs were placed at a distance from a d.c magnet, directly underneath the culture plate. The distance of magnet from cell culture plate needed to create 100 Oe field was determined using a gauss meter. After the end of incubation period, the cells were washed with phosphate buffered saline (PBS) to remove particles not strongly bound to cells. Cells were scraped from the bottom of plate and transferred to a 5 mm NMR tube. Continuous wave- ^1^H NMR spectra were obtained using a continuous wave (CW) spectroscope. Sample placement, instrument parameters (B0, instrument phase, line-width) were carefully selected to ensure optimal signal to noise ratio. Each NMR spectrum was collected at opposite phases (in our case these were 107 and 297). Signal processing such as solvent suppression, baseline correction and inverse fourier transform were performed in MATLAB® (Mathworks, MA)

### Atomic force microscopy imaging

U-87 MG and Brain endothelial cells were grown on poly-l-lysine coated cover slips and were incubated with MENs similarly as described above. After the incubation, the coverslips were washed with phosphate buffered saline (PBS) and were transferred to stubs. The live cell/wet atomic force microscopy (AFM) mode of a Multimode was used to obtain AFM images of cells with Bruker AFM probes DNP-S10 using a three-port electrochemistry tapping fluid cell element ECFC. The cantilever C had a resonant frequency of 56 kHz in air and spring constant of 0.24 N/m. In the engaged mode, the frequency dropped to approximately 8 kHz. The AFM scans were performed at a rate ranging from 1.4 to 2.6 Hz, a scan size on the order of a few microns, a Z-range of 50 nm and a Z-range phase of 10 degrees.

### Vibrating sample magnetometry

A room-temperature Lakeshore vibrating sample magnetometer (VSM) with a 3-T magnetic field sweep was used to measure key magnetic properties of nanoparticles under study including the magnetization saturation and the magnetic coercivity. A cryogenic VSM Quantum Design PPMS with a 9-T superconducting magnet was used to measure M-H temperatures in a wide temperature range, from 4 K to over 300 K.

### Transmission electron microscopy

Phillips CM-200 200 kV Transmission Electron Microscope (TEM) with Energy Dispersive Spectroscopy (EDS) option was used to obtain TEM images and EDS profiles.

## Conclusion

Multiferroic core-shell magnetoelectric nanoparticles with a diameter of 30 nm’s were formulated using the solvothermal route. Magnetic and electric fields are intrinsically coupled in this system. Particle adsorption on the cell membrane was dependent on the membrane morphology, which was based on the cell type as observed under live-cell AFM imaging. Due to the distinct association with cells and the ME effect the NMR adsorption spectra for cells incubated with MENs was significantly different compared to cells without any MENs. In contrast, conventional magnetic nanoparticles caused only a minor or no change in the adsorption spectra. We conclude that the minimization of ME energy on particle binding with the cells is responsible for the change in NMR adsorption spectrum in case of MENs. Using MENs as NMR sensitive probes adds another dimension in addition to the existing magnetic diagnostic probes for cancer diagnostics.

## Electronic supplementary material

Supplementary Information
