# Erratum: Multiferroic coreshell magnetoelectric nanoparticles as NMR sensitive nanoprobes for cancer cell detection

**DOI:** 10.1038/s41598-017-12311-9

**Published:** 2017-10-26

**Authors:** Abhignyan Nagesetti, Alexandra Rodzinski, Emmanuel Stimphil, Tiffanie Stewart, Chooda Khanal, Ping Wang, Rakesh Guduru, Ping Liang, Irina Agoulnik, Jeffrey Horstmyer, Sakhrat Khizroev

**Affiliations:** 1 Department of Electrical Engineering Florida International University, Miami, Florida 33174 USA; 2 Herbert Wertheim College of Medicine, Florida International University, Miami, Florida 33199 USA; 3 Electrical and Computer Engineering, University of California, Riverside, CA 92506 USA; 4 Neuroscience Centers of Florida Foundation, Miami, FL 33133 USA

*Scientific Reports* **7**:1610; doi: 10.1038/s41598-017-01647-x; Article published online 03 May 2017

The original version of this Article contained an error in the author list where the authors Tiffanie Stewart and Chooda Khanal were inadvertently combined.

The affiliations of Tiffanie Stewart and Chooda Khanal are:

“Department of Electrical Engineering Florida International University, Miami, Florida, 33174, USA”

This has now been corrected in the HTML and PDF versions of this Article.

