# Supplementary material for: Multiferroic coreshell magnetoelectric nanoparticles as NMR sensitive nanoprobes for cancer cell detection
Source: Sci Rep. 2017 May 9;7:1610. doi: 10.1038/s41598-017-01647-x (PMC5431629; doi:10.1038/s41598-017-01647-x)
Supplement: Supplementary file 1 — Supplementary Information [file 41598_2017_1647_MOESM1_ESM.pdf]

Supplementary Information

Multiferroic coreshell magnetoelectric nanoparticles as NMR sensitive nanoprobes for cancer cell detection

Abhignyan Nagesetti[a], Alexandra Rodzinski[b], Emmanuel Stimphil[a], Tiffanie Stewart, Chooda Khanal[a], Ping Wang[a], Rakesh Guduru[a], Ping Liang[c], Irina Agoulnik[b], Jeffrey Horstmyer[d], and Sakhrat Khizroev[a,b]*

[a] Department of Electrical Engineering Florida International University, Miami Florida, U.S.A 33174.

[b] Herbert Wertheim College of Medicine, Florida International University, Miami, Florida, U.S.A 33199.

[c] Electrical and Computer Engineering, University of California, Riverside, CA 92506.

[d] Neuroscience Centers of Florida Foundation, Miami, FL 33133

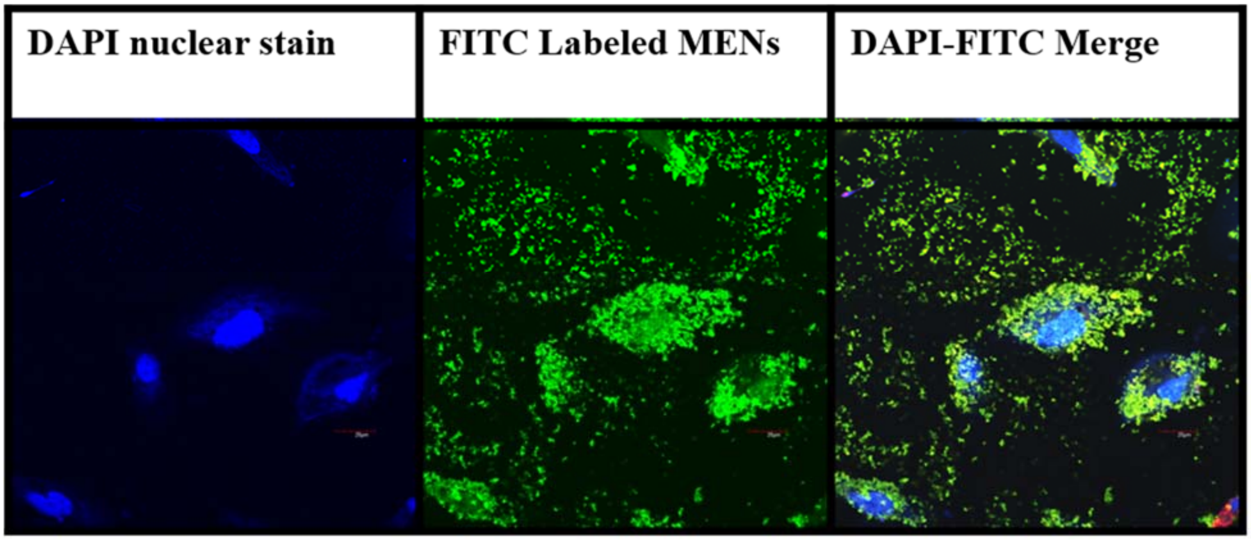

S1: Confocal Images of Glioblastoma cells incubated with Fluorescein Isothiocyanate (FITC) labelled MENs for 8 hours. Localization of particles with the cells can be clearly observed. Scale bar is 20µm.
